# Fuzzy logic selection as a new reliable tool to identify molecular grade signatures in breast cancer – the INNODIAG study

**DOI:** 10.1186/s12920-015-0077-1

**Published:** 2015-02-07

**Authors:** Tatiana Kempowsky-Hamon, Carine Valle, Magali Lacroix-Triki, Lyamine Hedjazi, Lidwine Trouilh, Sophie Lamarre, Delphine Labourdette, Laurence Roger, Loubna Mhamdi, Florence Dalenc, Thomas Filleron, Gilles Favre, Jean-Marie François, Marie-Véronique Le Lann, Véronique Anton-Leberre

**Affiliations:** CNRS, LAAS, F-31400 Toulouse, France; Université de Toulouse; INSA, UPS, INP; LISBP, F-31077 Toulouse, France; INRA, UMR792, Ingénierie des Systèmes Biologiques et des Procédés, F-31400 Toulouse, France; CNRS, UMR5504, F-31400 Toulouse, France; Institut Claudius Regaud, Biology and Pathology Department; INSERM UMR1037, Toulouse, France; Dendris SAS, 8 Rue de Cugnaux, 31300 Toulouse, France; Institut Claudius Regaud, Oncology Department, Toulouse, France

**Keywords:** Breast cancer, Molecular grade, Gene signatures, Fuzzy logic

## Abstract

**Background:**

Personalized medicine has become a priority in breast cancer patient management. In addition to the routinely used clinicopathological characteristics, clinicians will have to face an increasing amount of data derived from tumor molecular profiling. The aims of this study were to develop a new gene selection method based on a fuzzy logic selection and classification algorithm, and to validate the gene signatures obtained on breast cancer patient cohorts.

**Methods:**

We analyzed data from four published gene expression datasets for breast carcinomas. We identified the best discriminating genes by comparing molecular expression profiles between histologic grade 1 and 3 tumors for each of the training datasets. The most pertinent probes were selected and used to define fuzzy molecular grade 1-like (good prognosis) and fuzzy molecular grade 3-like (poor prognosis) profiles. To evaluate the prognostic performance of the fuzzy grade signatures in breast cancer tumors, a Kaplan-Meier analysis was conducted to compare the relapse-free survival deduced from histologic grade and fuzzy molecular grade classification.

**Results:**

We applied the fuzzy logic selection on breast cancer databases and obtained four new gene signatures. Analysis in the training public sets showed good performance of these gene signatures for grade (sensitivity from 90% to 95%, specificity 67% to 93%). To validate these gene signatures, we designed probes on custom microarrays and tested them on 150 invasive breast carcinomas. Good performance was obtained with an error rate of less than 10%. For one gene signature, among 74 histologic grade 3 and 18 grade 1 tumors, 88 cases (96%) were correctly assigned. Interestingly histologic grade 2 tumors (n = 58) were split in these two molecular grade categories.

**Conclusion:**

We confirmed the use of fuzzy logic selection as a new tool to identify gene signatures with good reliability and increased classification power. This method based on artificial intelligence algorithms was successfully applied to breast cancers molecular grade classification allowing histologic grade 2 classification into grade 1 and grade 2 like to improve patients prognosis. It opens the way to further development for identification of new biomarker combinations in other applications such as prediction of treatment response.

**Electronic supplementary material:**

The online version of this article (doi:10.1186/s12920-015-0077-1) contains supplementary material, which is available to authorized users.

## Background

Breast cancer, the most common invasive cancer in women, is an heterogeneous and complex disease. Currently, the management of breast cancer patients is based on clinicopathological characteristics such as age, menopausal status, tumor size, lymph node status, histologic grade [1] and on three immunohistochemical predictive markers: estrogen (ER) and progesterone (PR) receptors and human epidermal growth factor receptor 2 (HER2) [2,3].

Clinicopathological parameters and immunohistochemical markers are combined in guidelines such as St Gallen’s consensus and Nottingham [4] prognostic Index or incorporated in internet algorithms such as Adjuvant!Online (https://www.adjuvantonline.com/index.jsp), for treatment decision making. The combination of these parameters provides assessments of the benefit of a systemic endocrine or chemo-therapy [5]. Although effective in the reduction of mortality, these principles have shown limits and are not sufficient for individualized medicine. Tumors with similar clinical characteristics can have noticeably different outcomes in terms of treatment response and survival. Furthermore, over-treatment with adjuvant therapy is not harmless [6].

For the past decade, genome-wide microarray-based expression profiling studies have been used as a powerful tool to improve understanding of the biology of breast cancer [7-12]. With these technologies, many prognostic gene expression signatures were identified to predict breast cancer recurrence risk [13-21]. Molecular differences even among tumors with similar pathological features have been unravelled and a new molecular taxonomy for breast cancer classification into several subgroups (luminal A and B, basal-like, HER2, normal breast-like) has been proposed. However, most of these gene signatures are still under development for prospective validation in clinical trials. Despite the promise of previous gene signatures, decisions making in clinical practice are still guided by traditional parameters. Integrating microarray information and using it as a complement to clinicopathological parameters could provide more accurate and robust prognostic tests in order to guide adjuvant systemic treatment that could reduce the cost of breast cancer treatment.

Biological data obtained from high throughput technologies (DNA microarray, NGS, and so forth) are known to generate certain level (amount) of imprecise and noisy data. Moreover, the high dimensionality of these technologies data (tens to hundreds of thousands of features) makes use of machine learning and data mining techniques necessary, since many of these features are irrelevant or redundant. Many research efforts have been directed in the last two decades towards developing efficient feature selection methods [22-25]. The existing methods are traditionally categorized as filter or wrapper methods, with respect to the criterion used to search for relevant features [26,27]. In filter approaches, features are scored and ranked according to a certain statistical criteria and those with the highest ranking values are selected. Most frequently used filter methods include *t-*test [28], chi-square [29], Wilcoxon [30], Pearson correlation coefficients [31] and Principal Component Analysis [32]. Filter methods are fast but lack in robustness against feature interactions and redundancy [33]. Besides, the way to determine the rankings cut-off point to select only truly important features and exclude noise is not clear. Wrapper methods use the performance of a learning method to assess the quality (accuracy) of the selected feature subset in predicting the target (e.g. determined by cross validation). Wrapper methods employ search algorithms to determine an optimal subset of features. The most generally used search approaches are backward elimination and forward selection [26]. Stochastic algorithms, developed for solving large scale combinatorial problems, such as ant colony optimization, genetic algorithms, and simulated annealing are also used [34-36]. Although these algorithms efficiently capture feature redundancy and interaction they are computationally expensive. Recently, some authors take advantage of both filter and wrapper methods and propose hybrid algorithms [33,37-39]. The idea is to apply first a filter method to select a feature pool and then the wrapper method is applied to determine the optimal subset of features from the selected pool. The most popular learning algorithm used in wrapper methods is the support vector machines (SVM) [40]. Nevertheless, the accuracy of an SVM depends on the choice of the parameters and the kernel function. SVMs are sensitive to noisy training data, which can degrade their performance. Even more, they are prone to over-fitting and poor generalization. The principal drawback of wrapper methods is the computational cost, since they evaluate the feature subset with a learning algorithm, which are usually iteratively. Therefore to enhance the wrapper approach performance it is necessary to use fast learning algorithms which performs well when dealing with noisy and imprecise data.

Fuzzy logic was introduced in 1965 by Zadeh [41], it deals with reasoning that is approximate rather than fixed and exact. Contrary to traditional Boolean logic, where objects are classified either true (1) or false (0), in fuzzy logic, they may have values ranking from 0 to 1. Fuzzy logic has been widely used in system control due to its simplicity and effectiveness, especially in the case of nonlinear and high-dimensional systems. This is mainly due to its fundamental concept which enables to handle and manipulate imprecise and noisy data. Additionally, it provides an intuitive interpretation of the results. Although some attempts to use fuzzy logic to perform feature selection have been proposed [42-49], certainly these methods perform well when dealing with imprecise and noisy data but they generally end up with a high sophistication. Either they depend on a specified method (e.g. Fuzzy C-Means [50]) which is designed originally for clustering, or they use an arbitrary choice to determine the linguistic terms of the “fuzzified” features, which is not always possible and accurate enough whenever a big number of features must be tackled. Moreover, in order to reduce computational cost, some authors have combined fuzzy selection mechanisms with genetic algorithms or even have introduced the concept of fuzzy entropy for selecting relevant features [44-46].

Fuzzy classifiers have recently shown their effectiveness in classification tasks, since they enable dealing with noisy and imprecise information which is often present in many applications. However, their performances decrease significantly in case of high dimensional and/or heterogeneous problems. Despite these drawbacks, an increasing interest in applying fuzzy classifiers to breast cancer prognosis, using gene expression data has been observed [51,52].

In contrast to previous studies where fuzzy logic was used for assessing patient classification, we herein developed feature selection and classification algorithms both based on the fuzzy logic concept of membership degree. Our approach involves feature weighting based on a membership margin in order to improve the performance of fuzzy classifiers on high dimensional and heterogeneous problems. The effectiveness of this method has been previously demonstrated on problems involving mixed type of data (numerical, qualitative, symbolic intervals) [53].

One of the most important parameter in breast cancer is the histological tumor grade which classifies patients in three classes: 1, 2 or 3. Histologic grade 1 and 3 statuses are related to a low and high risk of recurrence, respectively. However, about 50% of tumors are classified as histologic grade 2, the moderately differentiated grade [13,14]. This grade is associated with an unclear risk of recurrence and is thus not informative for clinical decision making. Individualized medicine, not only based on clinicopathological characteristics, but also using information derived from tumor molecular profiles could improve patient management and increase survival. However it remains a clinical challenge. We have applied this new fuzzy logic methodology on gene expression data information obtained from public available datasets and our own patients cohort. We identified gene signatures enabling accurate discrimination of patients with breast cancer into either grade 1 or grade 3 and to help classifying patients with histologic grade 2 tumors into grade 1 or grade 3 like. This classification could lead to a reduction of over- and under- treated patients.

## Results

### Gene signatures according to *fuzzy* molecular grade (*f*MG)

**Figure 1:**
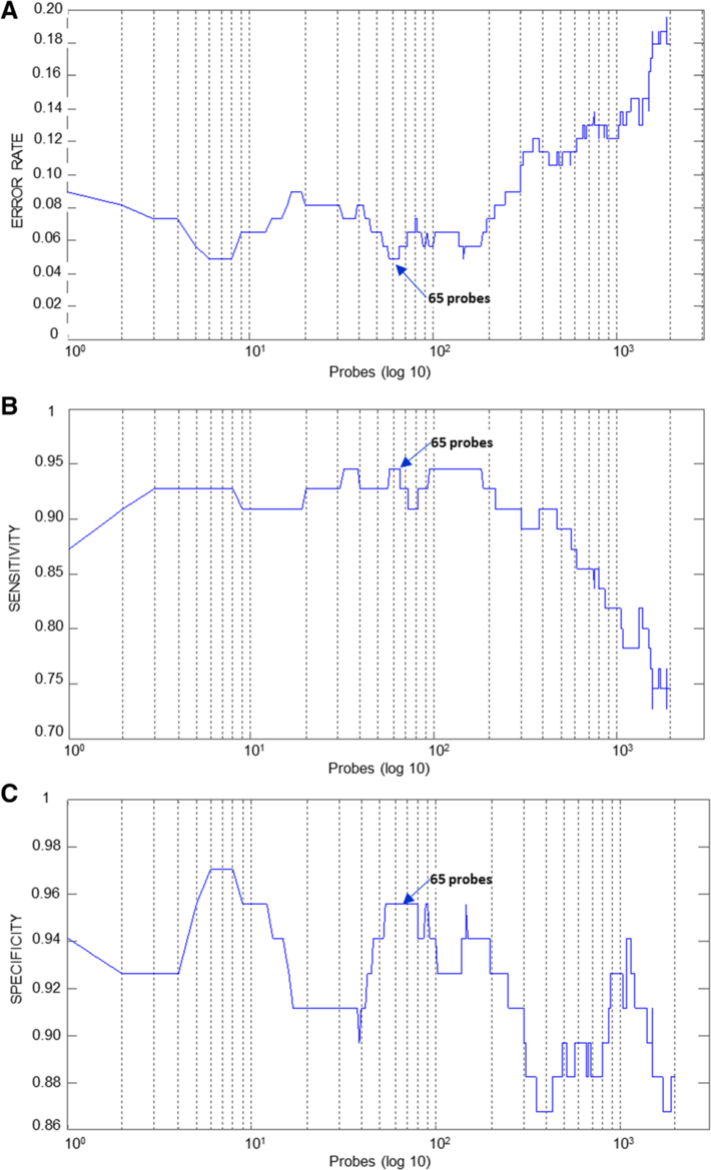
**Selection of the most discriminant probes for GS C – GSE4922** **[** 13 **]** **.** Minimum number of probes providing the best sensitivity with a low global error. **A)** Global Error, **B)** Sensitivity, **C)** Specificity. The number of evaluated probes is expressed in a log 10 scale (horizontal axis).

We applied the fuzzy logic selection algorithm MEMBAS on four public available cohorts to identify prognostic gene expression signatures for breast cancer, based on histologic grade. The use of several cohorts allowed taking into account a maximum heterogeneity of patients. Due to the different microarray platforms (Affymetrix, Agilent Technologies), experimental protocols and data processing for normalization used by authors, we choose to use gene expression raw data individually. In the training sets, we analyzed expression profiles of 452 patients with primary breast cancer with histologic grade 1 or 3. Histologic grade 2 patients were excluded since our objective was to identify low and high risk profiles in order to classify later intermediate grade tumors as molecular grade 1-like (*f*MG1) or 3-like (*f*MG3). Tumor data were derived from four independent cohorts: NKI2-Agilent (113 samples) [16], KJX64KJ125-GSE2990 (103) [14], Uppsala-GSE4922 (123) [13], and Transbig-GSE7390 (113) [6]. The training sets were used to identify groups of genes whose expression allowed distinguishing histologic grade 1 from grade 3 tumors. MEMBAS algorithm ranked in decreasing order the probes and a posterior probability was iteratively estimated for each tumor by leave-one-out cross validation (LOOCV). We performed selection of the minimal number of probes which achieved the highest possible sensitivity (Histologic Grade HG3 patients correctly identified) and an acceptable level of specificity (HG1 patients correctly classified), in order to have accurate grade assignments (Figure 1). In breast cancer context, a high sensitivity is privileged over high specificity in order to be sure that patients with high risk of recurrence would be thoroughly treated.

We identified 67 Agilent probes and 38 Affymetrix probes respectively for the gene signatures A and B, designed from only ER positive patients of NKI2 and KJX64-KJ125 cohorts; whereas gene signatures C and D were composed of respectively, 71 and 18 Affymetrix probes selected on both ER positive and negative tumors for Uppsala and Transbig cohorts. These probe sets correspond respectively to 65, 37, 65 and 16 unique genes respectively. Most genes were overexpressed in grade 3 tumors. As highlighted by molecular taxonomy, ER positive and ER negative breast cancers are fundamentally different diseases and ER status may influence gene expressions. For this reason, we tested ahead stratification of tumors, according to their ER status (either only ER positive or both ER positive and negative) and selected the gene signatures with the best discriminant power for each cohort, regardless of ER status for patients included in the training set.

When we compared the new gene signatures obtained with our fuzzy logic strategy with the previously published prognostic signatures, we found several genes in common (Additional file 1: Table S1). For example in gene signature C [13], 14 of the 18 genes from the PAM genetic grade signature [13] were identified. More than 77% of the genes selected in Affymetrix gene signatures B [14], C [13] and D [6] were common to Sotiriou’s GGI signature [14].

### Grade 1&3 profiles - training set

We examined the performance of our gene signatures in predicting histologic grade 1 and 3 by determining whether the classification obtained by this molecular method agrees with the histologic ones. Sensitivity and specificity rates of each signature on the respective training sets are summarized in Table 1. Best results were obtained with gene signature C on Uppsala cohort [13]. Only 3 out of 55 histologic grade 3 tumors (5%) were classified in molecular grade 1-like and 5 out of 68 histologic grade 1 tumors (7%) were assigned to molecular grade 3-like. Secondly, gene signatures A [16] and B [14] achieved a sensitivity of 90% (5/49 and 4/40 misclassifications of grade 3) and a specificity of 86% and 87% respectively (9/64 and 8/63 misclassifications of grade 1). Gene signature D [6] presented a high rate of sensitivity (93%) and a lower specificity (67%), likely due to an imbalance in the composition of grade 1 and 3 tumors in this cohort and to a lower number of grade 1 tumors. To sum up, cancer grades prediction obtained from the four signatures strongly correlated with those obtained from histologic data.

**Table 1:** **Classification agreement between molecular (** ***f*** **MG) and histologic grades (HG) in training cohorts**

				**Histologic grade**					
**Gene signature**	**Training cohort**	**Total patiens (N)**	**Molecular grade (** ***f*** **MG)**	**HG1**		**HG3**		**HG2**	
				**n**	**%**	**n**	**%**	**n**	**%**
*fGS* A	NKI2 [16]	206	G1	**55**	**(86)**	5	(10)	46	(49)
			G3	9	(14)	**44**	**(90)**	47	(51)
*fGS* B	KJX64-KJ125 [14]	166	G1	**55**	**(87)**	4	(10)	38	(60)
			G3	8	(13)	**36**	**(90)**	25	(40)
*fGS* C	Uppsala [13]	249	G1	**63**	**(93)**	3	(5)	82	(65)
			G3	5	(7)	**52**	**(95)**	44	(35)
*fGS* D	TVBDX [6]	196	G1	**20**	**(67)**	6	(7)	32	(39)
			G3	10	(33)	**77**	**(93)**	51	(61)

% in bold correspond to specificity (HG1) and sensibility (HG3).

**Figure 2:**
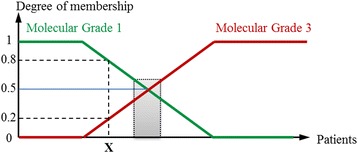
**Membership degree of a patient X to both molecular grade 1(green) and grade 3 (red) classes.** The gray rectangle, for membership values around 0.5, corresponds to an uncertainty zone where gene expression values are intermediate.

As detailed in the Methods section (Figure 2), to quantify the degree of membership to molecular grade 1-like (*f*MG1) and 3-like (*f*MG3), a molecular grade score was developed. As shown in Figure 2, a tumor displaying a grade 3-like gene expression profile is assigned with a score (membership degree) greater than or equal to 0.5, whereas a score below 0.5 corresponded to a tumor displaying a grade 1-like gene expression profile. The values of molecular grade score for all datasets are shown in Figure 2.

**Figure 3:**
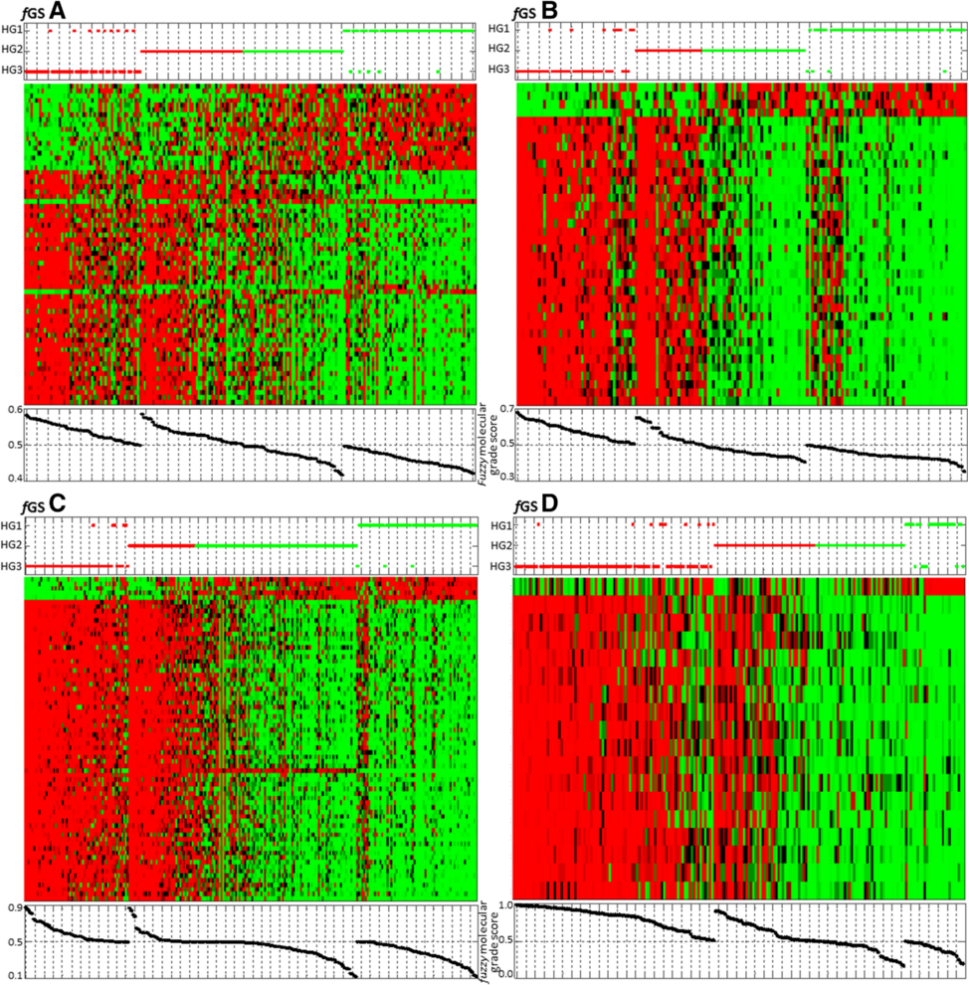
**Heat maps for fGS A, B, C and D in their corresponding training sets** **[** 16 **,** 14 **,** 13 **,** 6 **]** **.** Each dataset was standardized along rows so that the mean is 0 and the standard deviation is 1. Red corresponds to positive expression values and green to negative expression values. Color intensity reflects the magnitude of expression relative to the mean. Rows correspond to gene probe sets, ranked in descending order (from bottom to top) according to MEMBAS feature selection algorithm. Columns of heat maps correspond to tumors, which were grouped according to their assigned molecular profile (LAMDA classification). TOP panel: Red dots = molecular grade 3 profile; green dots = molecular grade 1 profile. Vertical axis corresponds to tumors’ histologic grade (HG3, HG2 and HG1 from bottom to top). BOTTOM panel: Molecular Grade score of each tumor is plotted below the corresponding column.

Molecular grade scores were well correlated to the gene expression patterns in Figure 3. The four gene signatures with molecular grade scores accurately classified grade 1 and 3 tumors with few misclassifications.

### Grade 2 classification - training set

To determine if we were able to separate histologic grade 2 tumors in two classes, i.e. grade 1-like and grade 3-like, we used the same classification algorithm (i.e. LAMDA) preserving the same profiles of histologic grade 1 and 3 tumors (Table 1). The gene expression profiles of the histologic grade 2 tumors were similar to either molecular grade 1 profile or molecular grade 3 profile (Figure 3). Thus Figure 3 shows that most grade 2 tumors can be molecularly separated either into grade 1-like or grade 3-like classes.

Although our fuzzy gene signatures could accurately distinguished grade 1 from grade 3 tumors and separated grade 2 tumors in grade 1 like or grade 3 like, we defined an indecision zone that scored arbitrarily between 48 and 52%, due to the equivocal gene expression grade profile of these patients (Figure 3 Heat map). For patients whose classification score lies within this uncertainty zone, their gene expression grade profile cannot be determined with certainty. Equivocal profiles represented a sizeable part of tumors (20 to 41%) according to the cohort, and this is particularly highlighted by the slope of the grade 2 molecular score on the *f*GS C (Figure 3, *f*GS C bottom panel). The origins of these intermediate tumors are unclear and could be biological or technical.

### Gene signature (GS) performance assessment in independent validation sets

**Figure 4:**
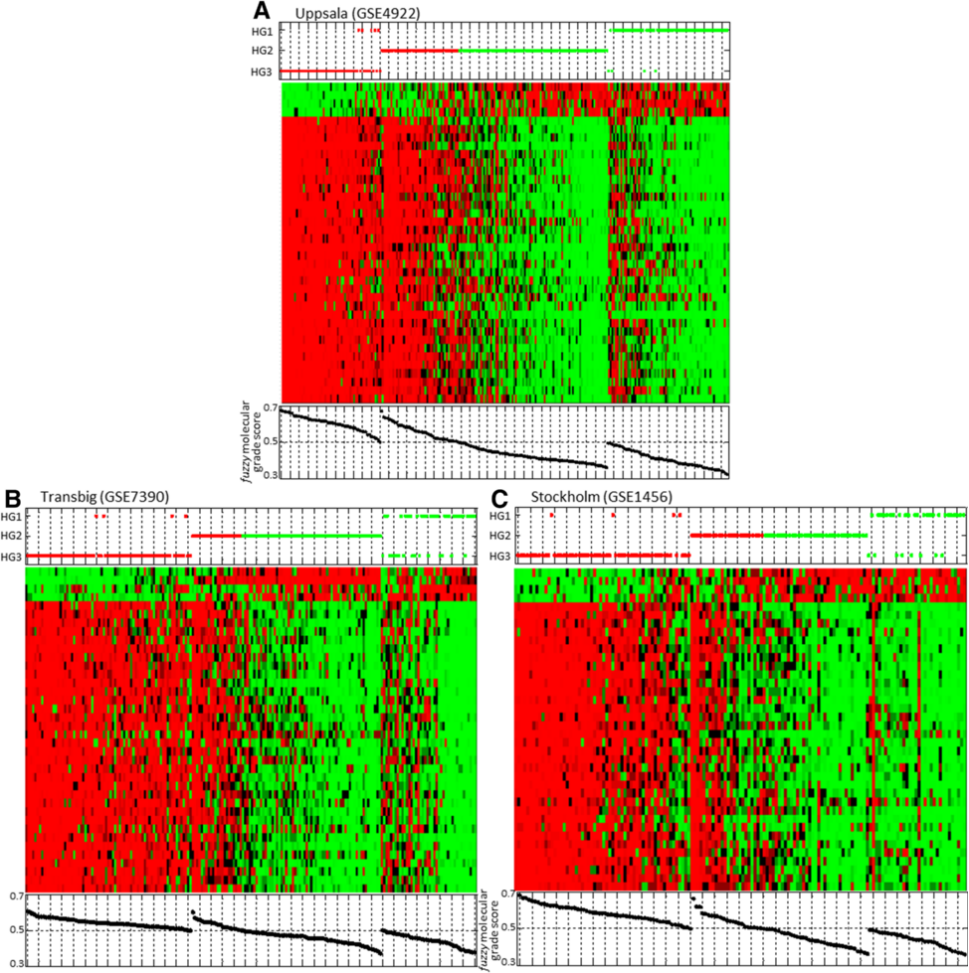
**Cross validation of fGS B. Genes of the fGS B were mapped to three previously published breast cancer microarray datasets: A) Uppsala (GSE4922)** **[** 13 **]** **; B) Transbig (GSE7390)** **[** 6 **]** **; and C) Stockholm (GSE1456)** **[** 13 **]** **.** HG1 and HG3 tumors from each dataset were used to calculate molecular grade profiles; HG2 tumors were classified as fMG 1-like or fMG 3-like (top panel) and sorted according to their molecular grade score (bottom panel).

An important aspect of our *fuzzy* gene signatures (*f*GS) is their performance. It has to be assessed on cohorts from various origins, including a validation set. Therefore, we tested the four fuzzy gene signatures (fGS) on several independent patient cohorts. We used exactly the same probes than the ones included in each *f*GS in order to strictly respect selected molecular profiles. Only gene signature A could not be validated on an independent dataset due to the deficiency of other Agilent platform datasets. Furthermore, redundant patients present in KJX64/KJ125 and Uppsala datasets were removed from the validation tests so they were only considered once. Validation tests showed that gene signatures B, C and D achieved similar performances to those obtained in training sets with about 80-90% of sensitivity (classification of HG3 tumors) and specificity (classification of HG1 tumors), and error rates between 7 to 17% (Additional file 2: Table S2). Figure 4 shows the cross validation *f*GS B on the different cohorts. We observed a suitable agreement in classification between molecular and histologic grades. The gene expression profiles of histologic grade 1 and 3 tumors for each gene signature were similar to those identified in the training set (Figure 3*f*GS B). We also found, as in the training set, close associations between the gene expression profiles, fuzzy molecular grade scores and histologic grades. We can notice that it exhibited good results with both ER positive and negative tumors although its fuzzy molecular grade score was constructed initially with only ER positive tumors to avoid bias. Furthermore, we calculated fuzzy molecular grade scores for grade 2 tumors in validation sets. We observed that, albeit an equivocal zone was observed, most grade 2 tumors could be separated in *f*MG 1-like and *f*MG 3-like (Figure 4).

### Correlation between fuzzy molecular grade score and relapse-free survival

To estimate the prognostic performance of gene signatures in breast cancer patients, the Kaplan-Meier method [54] was used to compare the relapse-free survival deduced from histologic classification to that from fuzzy molecular classification.

The reliability of molecular grades in the different datasets and in pooled datasets was tested. Histologic grade 3 tumors were associated with a high rate of relapse whereas histologic grade 1 tumors were related to a low risk of recurrence. Histologic grade 2 tumors were associated with an intermediate rate of relapse. We performed the same survival analysis with fuzzy molecular grade obtained with the four gene signatures. The results summarized by hazard ratios, 95% confidence intervals and p value, are presented in Table 2. The hazard ratio from all datasets classified with the four gene signatures showed significant log rank test p value. Only gene signature D failed to reach significance in its training set (Transbig cohort), however histologic grading did too. As shown in Table 2, discriminating grade 1 from grade 3 tumors with our Fuzzy molecular method exhibited better results than with the histologic one. In addition, while survival curves of molecular grade 1 and 3 were similar to those of histologic grade 1 and 3 respectively, in some cases, fuzzy gene signatures could be better than the histologic grade classification in improving relapse free survival for patients with grade 1 and 3 tumors (Additional file 3: Figure S1).

**Table 2:** **Survival analysis of grade 1 and 3 tumors classified with fuzzy molecular and histologic grades**

***fuzzy*** **Gene Signature**	**HG 1&3 patients**	***fuzzy*** **Gene Signatures**		**Histologic grade**	
	**n**	**Hazard Ratio (95% CI)**	**p value logrank test**	**Hazard Ratio (95% CI)**	**p value logrank test**
***f*** **MG A**					
NKI2 training	113	2.136 (1.466 - 3.113)	<.001	1.5896 (1.135 - 2.213)	<0.0052
NKI2 validation all	163	1.989 (1.463 - 2.704)	<.001	1.694 (1.294 - 2.218)	<.001
***f*** **MG B**					
KJX64/KJ125 training	99	1.923 (1.342 - 2.754)	<.001	1.546 (1.075 - 2.223)	<0.0184
Transbig	113	1.435 (1.005 - 2.051)	<0.0426	1.062 (0.792 - 1.426)	=0.0541
Stockholm	89	2.711 (1.313 - 5.595)	<0.00158	2.104 (1.28 - 3.459)	<0.0103
Pool 1	272	1.59 (1.25 to 2,02)	<0.0001	1.55 (1.20 to 2.00)	<0.001
***f*** **MG C**					
Uppsala training	123	1.484 (1.091 - 2.017)	<0.0103	1.773 (1.306 - 2.408)	<.001
Stockholm	89	4.134 (1.518 - 11.258)	<.001	2.104 (1.28 - 3.459)	<0.0103
***f*** **MG D**					
Transbig Training	113	1.28 (0.852 - 1.923)	=0.23	1.062 (0.792 - 1.426)	=0.0541
KJX64/KJ125	122	1.591 (1.157 - 2.186)	<0.00312	1.518 (1.11 - 2.077)	<0.00725
Stockholm	89	3.844 (1.412 - 10.469)	<.001	2.104 (1.28 - 3.459)	<0.0103
Pool 2	281	1.65 (1.31 to 2,07) supp KJX	<0.00001	1.66 (1.32 to 2.09)	<0.00001

Pool 1: Uppsala + Transbig + Stockholm.

Pool 2: KJX64/KJ125 + Uppsala + Stockholm.

**Figure 5:**
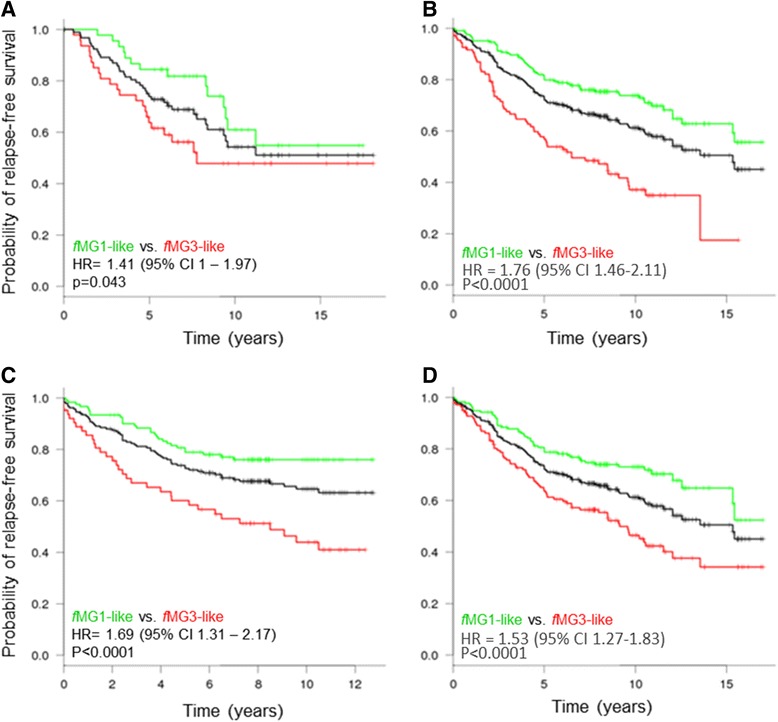
**Relapse free survival analysis of patients with histologic grade 2 tumors (black) classified in fMG1-like (green) and fMG3-like (red) by fuzzy Gene Signatures (fGS).** Hazard ratios with 95% confidence intervals (CI) and log-rank test (p value) were calculated to evaluate significance (fMG1-like vs. fMG3-like). **(A)** For fGS A, NKI2 cohort was used (n = 93). **(B, D)** For fGS B and D respectively, Kaplan-Meier analysis were conducted with pooled data of KJX64/KJ125, Uppsala, Stockholm, Transbig cohorts (n = 309). **(C)** For fGS C, Uppsala and Stockholm cohorts were pooled (n = 184).

To evaluate the clinical interest of molecular grades, grade 1-like and grade 3-like, we compared the relapse-free survival of grade 2 patients who had grade 1-like profile with that of patients who had grade 3-like profile, according to the four gene signatures. The four fuzzy molecular grade signatures separated grade 2 tumors into 2 distinguished classes, grade 1-like and grade 3-like, with a statistically significant difference in relapse-free survival across all datasets or when datasets were pooled (Figure 5 and Additional file 4: Table S3). Grade 2 tumors with grade 1-like profile had a lower risk of recurrence than grade 3-like.

We examined several variables in a univariate analysis (Additional file 5: Table S4) and found that gene expression grade of our *f*GS, histologic grade, lymphoma node status and tumor size were all statistically significantly associated with relapse-free survival. However, in a multivariable analysis, only our fuzzy Gene Signature (*f*GS) and tumor size remained statistically significant associated with relapse-free survival. Our *f*GS having the strongest association: HR = 1.51, 95% CI = 1.21 to 1.88; P < 0.0004 .

### *Fuzzy* molecular grade (*f*MG) validation on an independent cohort

#### ICR cohort on Nimblegen custom microarray

In order to evaluate the performance of our four gene signatures, we designed a NimbleGen custom microarray with the genes obtained on our gene signatures and validated them thanks to expression profiles of breast cancer tumors from a new cohort (C. Regaud Institute, n = 150). This cohort consists of 18 histologic grade 1 tumors (11.9%), 58 (38.7%) grade 2 and 74 (49.3%) grade 3 tumors. The custom NimbleGen array was composed of genes of interest with 9 probes for each transcript. We used MEMBAS selection algorithm, instead of the conventional mean value, in order to rank (descending order) all probes representing the genes included in a gene signature. We selected the best ranked probe for each gene (Additional file 5: Table S4). Fuzzy molecular grade 1 and 3 profiles were determined with these selections and with LAMDA classification algorithm.

### Grade 1 & 3 profiles

**Figure 6:**
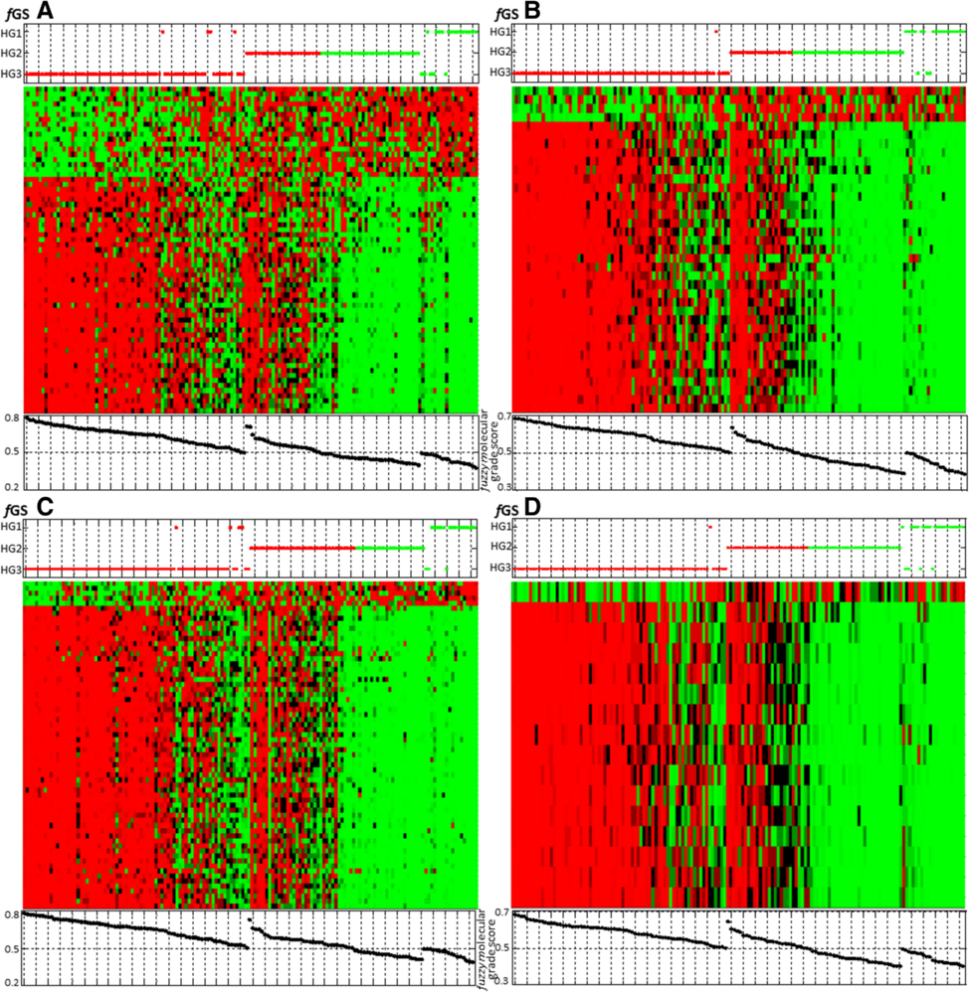
**Heat maps for fuzzy gene signatures A, B, C and D in the validation set (ICR): for each fGS, HG1 and HG3 tumors were used to calculate molecular grade profiles; HG2 tumors were classified as fMG 1-like or fMG 3-like (top panel) and sorted according to their fuzzy molecular grade 3 score (bottom panel).**

With Nimblegen microarrays constructed with the newly designed probes based on our *fuzzy* gene selection as described in Material and methods, we examined the expression profiles on ICRs cohorts for consistency with predicted histologic grade. As shown in Figure 6, the gene expression patterns of patients with histologic grade 1 (n = 18) and grade 3 (n = 74) tumors were similar to those identified previously in the training and validation sets from public datasets.

A total of 125 probe sets (representing 122 genes) were identified as genes with the highest discriminating power (i.e. the most significantly differentially expressed genes) between grade 1 and grade 3 tumors.

For all fuzzy gene signatures, we could easily discriminate low and high grade from the gene expression patterns of the ICR cohort. The accuracy of fuzzy molecular grading in terms of classified in low and high grade (*f*MG1 and *f*MG3) was evaluated using a LOOCV, since the number of grade 1 tumors is very small (11.9%) compared to the number of grade 3 tumors (49.3%). Results for high sensitivity and specificity are shown in Additional file 6: Table S5. For example, using fGS B, only one of the 18 HG 1 tumors (6%) showed a fuzzy molecular grade score greater than 0.5, and only three of the 74 HG 3 tumors (4%) displayed a fuzzy molecular grade score inferior to 0.5.

Most importantly, we noticed that misclassifications between gene signatures often corresponded to the same tumors. Indeed, in all four gene signatures, four tumors consistently showed an opposite molecular grade as compared to their histologic grade. Other tumor misclassifications were obtained in two or only one gene signature. Several of these tumors presented clinicopathological features that could explain these problems of concordance. Among the misclassified histologic grade 3 tumors, two tumors were invasive lobular carcinoma, of pleomorphic subtype (cases #37 and 113). One tumor corresponded to a heterogeneous tumor (as of micropapillary and IDC-NST histologic subtypes) (case #109). Another tumor displayed a triple negative phenotype (case #127). Two misclassified histologic grade 1 tumors were heterogeneous tumors with distinct components (cases #6 and 96), easily identified by microscopic examination (one case harboring a mucinous component, the one measuring 23mm of greater size).

### Grade 2 classification in *f*MG1-like or *f*MG3-like categories

After checking the accuracy of the *fuzzy* prediction model on grade 1 and 3 tumors with the new design of probes, we tested the profiles of histologic grade 2 tumors (n = 58). As for the public datasets, the histologic grade 2 tumors harbored extreme values than encompassed those of histologic grade 1 and 3 tumors (Figure 6). It could be noticed that 69% of grade 2 tumors were classified identically by the four gene signatures. Only 7% (4/58 tumors) of grade 2 tumors were ambiguously classified as *f*MG1-like by two gene signatures and as *f*MG3-like by the two others. Among these 4 tumors, 3 were within the equivocal zone. This demonstrates that the use of several gene signatures may prove useful to enhance confidence on prognostic information provided by molecular grade.

## Discussion

It has been shown that breast cancer histologic grade provides an important prognosis information, with grade 1 tumors showing a low risk of recurrence as opposed to grade 3 tumors harboring a high risk of recurrence. However, about 50% of breast cancers are classified as moderately differentiated (intermediate) histologic grade 2, which gives no information about the clinical decision or the treatment strategy to apply. Microarray gene expression data has been employed in order to increase the prognostic value of tumor grading, on the one hand by refining grade 2 tumors into two distinct categories which could be eventually related to low and high risk, and on the other hand improving reproducibility. In contrast to what has already been done, we have herein used a feature selection algorithm and a classifier based on fuzzy logic theory and concepts, in order to identify genes that best discriminate grade 1 from grade 3 tumors. We applied this approach to four previously published microarray datasets (Agilent and Affymetrix technologies) and generated four distinct *fuzzy* gene signatures. We found that all four grade signatures were able to reliably identify histologic grade 1 and 3 tumors and molecularly separate grade 2 tumors into *f*MG1-like or *f*MG3-like categories, regardless of the platform technology, experimental protocols or type of cohorts. The fact of applying the membership concept (i.e. a sample belongs simultaneously to each category with a given value of confidence) allowed representing the heterogeneity of tumors without the necessity of preprocessing into molecular subtypes. Hence, only those probes that truly contributed to the characterization of both grade 1 and grade 3 categories were selected. Moreover, the proposed fuzzy gene signatures succeeded to extend good classification results in other populations. The results were reproducible and gene signatures were validated across cohorts.

### Identified genes from Fuzzy GSs

As expected, the majority of genes selected with fuzzy logic encompassed genes involved in cell cycle control and proliferation. Only one gene, CENPA, was common to all four GS. The centromere protein A (CENP-A) is an essential centromere protein, required for chromosome segregation during cell division. CENP-A has been associated with high grade cancers and is a strong prognostic marker for distant relapse in ER-positive breast cancer. McGovern SL and their collaborators [55] demonstrated a clear relationship between the degree of expression of this essential protein and outcome in ER-positive breast cancer. Seven genes – AURKA, CDCA8, DDX39A, FOXM1, KIF2C, MELK, MKI67- were common to GS B, C and D (Affymetrix technology) (see Additional file 7: Table S6 and Venn Diagram Additional file 8: Figure S2). From the 122 selected genes, 46 of them are unique to GS A (Agilent), and 10, 23 and 1 are unique to GS B, C and D respectively. For GS A and B, generated with ER positive samples only, 7 genes overlapped (CENPA, BIRC5, CCNB1, KIF20A, KPNA2, RACGAP1).

Comparison of our four GS (122 selected genes) with previously published genetic grade signatures revealed a significant degree of overlap, in particular with PAM (Ivshina *et al.*) and GGI (Sotiriou *et al.*), where for some GS more than 77% of the genes overlapped. These findings not only confirm the good performance of our approach (in terms of biological coherence) but also reinforce the fact that gene expression-based profiles of histologic grade can contribute to patient prognosis identification.

### Equivocal cases

Although gene signatures accurately classify grade 1 and 3 tumors and separate grade 2 tumors in grade 1-like or grade 3-like, we noticed an uncertainty zone where molecular profiles were intermediate. This uncertainty zone (see Methods Figure 2) was confirmed by the molecular grade score value. In fact, for tumors displaying a score around 0.5, their molecular profile did not show a clear similarity neither to grade 3 nor to grade 1 profiles. Whilst it has been recently advocated, no consensual definition of this uncertainty zone is currently available in the literature. Arbitrarily, we defined as *equivocal* those cases with a classification score between 0.48 and 0.52. For patients whose classification score lies within this uncertainty zone, their gene expression grade profile cannot be distinctly determined as grade 1-like or grade 3-like.

When cross validation of our fuzzy gene signatures was performed on public cohorts, equivocal profiles (all histologic grades included) represented a more or less significant part of tumors, ranging from 12 to 31.3% depending on the cohort. Albeit the origins of these intermediate tumors remain unclear and debatable, it most likely corresponds to a true biological process, reflecting the spectrum and continuum of disease found in ER+ breast cancer, especially with regards to proliferation. Some of these equivocal cases could also represent heterogeneous tumors with a mixture of grade 1 cells and grade 3 cells.

Likewise, concerning the results obtained when applying our gene signatures to the ICR cohort we noted that gene signatures could disagree for some tumors in the assignment of histologic grade 2 into grade 1-like or grade 3-like. Thus, we investigated whether problematic histologic grade 2 tumors had extreme or intermediate profiles. We analyzed the range of scores of grade 2 tumors and we observed a continuous score slope as for grade 1 and 3 tumors. Furthermore, we noticed that gene signatures produced about 9% of equivocal profiles (between 7 to 11% of the cases depending on the gene signature). Whatever the grade gene signature, an intermediate zone thus exists. Most of grade 2 tumors classified differently by several gene signatures also corresponded to equivocal cases (their classification score ranging between 0.48 and 0.52).

## Conclusion

In conclusion, our approach of fuzzy genetic molecular grading allows refinement of histologic grade 2 tumors into *f*MG1-like and *f*MG3-like categories, although an uncertainty zone still remains. This suggests that genetic grade could be used, at some degree and at the very best in combination to clinicopathological parameters, to further improve outcome prediction of patients that should be spared of systemic adjuvant therapy. It seems that one unique Gene Signature might not be sufficient enough for decision making. Several gene signatures might thus be used to enhance confidence on prognosis provided by molecular grade. Moreover, the use of both clinicopathological characteristics and information derived from tumor molecular profiles could improve patient management and increase survival. It is noteworthy that our fuzzy feature selection algorithm and classifier is capable of treating features of different types simultaneously. The next challenge will be to include relevant clinicopathological characteristics into our fuzzy molecular grade signatures. Future works will be therefore oriented in evaluating our *f*GS performance in association to other parameters, in independent and larger cohorts.

Finally, we have demonstrated here the proof of concept of using fuzzy logic to select relevant biomarkers and to better evaluate risk. Individualized medicine remains a clinical challenge and will still need new prognostic and predictive biomarkers. The fuzzy logic method might prove of useful value in discovering new biomarker combinations in other main applications such as prediction of treatment response.

## Methods

### Breast microarray datasets

We collected four public available datasets from patients with primary breast cancer profiled using Affymetrix or Agilent DNA microarrays: the Netherlands Cancer Institute NKI2 dataset from van de Vijver *et al.* (256 patients), the KJX64/KJ125 datasets from Sotiriou *et al*. (189 patients), Uppsala dataset from Ivshina *et al.* (249 patients) and the Bordet Institute TRANSBIG dataset from Desmedt *et al*. (198 patients). Some samples of the NKI2 cohorts were excluded from our study due to missing or biased data. Redundant patients (74 samples) present in KJX64/KJ125 and Uppsala datasets were removed from the validation tests so they were only considered once. Gene expression and clinical data of public series were retrieved from Gene Expression Omnibus (GEO) public database http://www.ncbi.nlm.nih.gov/geo, author’s website and publications [6,13,14,16]. All datasets were retrospective. They are described in Additional file 9: Table S7.

### Selection of grade gene signatures and class prediction

To evaluate cancer recurrence risk, we identified gene signatures using information on the histologic grade. The aim was to classify grade 2 tumors with unknown outcome, into two subclasses: a grade 1-like subgroup with good outcome and a grade 3-like subgroup with poor outcome. For this, a feature selection technique and a classification algorithm based on fuzzy logic concepts were used.

### Fuzzy feature partition

Fuzzy set theory was proposed by Zadeh in 1965 to mathematically model the imprecision inherent to some concepts [41]. In short, fuzzy sets theory allows an object to partially (simultaneously) belong to a set (class) with a certain degree of membership between 0 and 1. In a machine learning framework, an approach is defined as “fuzzy” if we consider that an individual belongs to each class with a certain degree of membership, unlike the “crisp” (“hard”) approaches where each individual is considered to belong only to one class [56]. Taking this in consideration, we can apply these concepts to the problem of classifying breast cancer patients according to their *fuzzy* molecular (gene expression) grade (*f*MG) profile corresponding to a grade 1-like profile (*f*MG1) and a grade 3-like profile (*f*MG3). Figure 2 shows a representation of the membership degrees associated to each class for a group of patients. Then, patient X, in the figure, has a membership degree of 0.8 for *f*MG1 and 0.2 for *f*MG3. An ambiguous (or uncertainty) zone can be defined around a membership degree value of 0.5, representing those patients for which a low or high risk profile cannot be clearly associated, i.e. their molecular grade profile is really intermediate (see “gray rectangle” on Figure 2).

### Grade-associated gene selection

#### Fuzzifying gene expression data

In order to represent all the features (gene expression values) of a sample (patient/tumor) by their memberships to a reference fuzzy partition (molecular grade 1-like & 3-like), we used membership functions denoted as *μ*_*k*_^*i*^ based on the *similarity* (or distance) semantics [57]. As stated by Medasani and Kim [58], no measures are available to evaluate the goodness or correctness of a given membership function, nevertheless the success of an algorithm depends on the membership functions used. Several functions should be used in order to select the one that gives the best performance for pattern recognition according to the type of data (see Additional file 10). In this work, the *fuzzy* extension of the binomial function and the Gaussian function were used [58]. The first one, works extremely well when the observations are grouped, after standardization (see eq.1), around 0 or 1, but may present instability or definition problems when data is concentrated around 0.5. The second one is commonly used when the volume of the observed data is important, since it is very likely to follow a Gaussian or semi-Gaussian distribution. Also, this function measures the proximity to an estimated center. In this way gene expression values are transformed into the membership space without any information loss and are ready to be used for both feature selection and classification.

#### Fuzzy feature selection algorithm - MEMBAS

As previously mentioned existing feature selection algorithms are traditionally characterized as wrappers or filters according to the criterion used to search the relevant features [27,28]. We have recently proposed a new feature selection algorithm, referred to as MEMBAS for MEmbership Margin Based Attribute Selection [59] which enables to process similarly the three data types (numerical, qualitative, interval) based on an appropriate mapping using fuzzy logic concepts. The algorithm measures simultaneously the contribution of each gene for each of the two classes (in our case, Molecular Grade1 & Grade3 tumors), in order to find the best discrimination. That is, it extracts the most pertinent markers since it is based on feature weighting according to the maximization of a membership margin. To avoid the heuristic search during the feature selection procedure, MEMBAS optimizes a membership margin based objective function by using classical optimization techniques providing an analytical solution [60].

### Class prediction - Fuzzy classification algorithm

The learning and classification algorithm, LAMDA (Learning Algorithm for Multivariable Data Analysis) [61] has been used to generate the fuzzy partition that best discriminates histologic grade 1 and 3 patients according to their gene expression profiles, as well as to determine the probes that best fit this partition. LAMDA is a fuzzy methodology of conceptual clustering and classification which is based on finding the global membership degree of a sample to an existing class, considering all the contributions of each feature. This contribution is called the *marginal adequacy degree* (MAD). The MADs are calculated by means of a membership function and they are then combined using “fuzzy mixed connectives” as aggregation operators in order to obtain the *global adequacy degree* (GAD) of an element to a class. Finally a sample (tumor/patient) will be assigned to the class for which its GAD is the highest [62]. In Hedjazi et al. [60] and his PhD manuscript [63] an extensive experimental study, including a comparison with known feature selection methods has been performed on several datasets presenting mixed-type and high-dimensional data. The experimental results in these works show that MEMBAS leads to a significant improvement of classification performance of LAMDA (fuzzy classifier) as well as other well-known classifiers (k-NN, SVM). Moreover, the combined fuzzy model MEMBAS/LAMDA works well in datasets with mixed-type data, since the same fuzzifying process (membership functions) is used for both feature selection and classification. This provides a similar processing for each feature type with minimal loss of information.

### *Fuzzy* molecular grade – gene signature strategy

For each breast cancer cohort we identified the most relevant genes using MEMBAS/LAMDA algorithms. The procedure was as follows:

1. The cohort database was separated into a training set (histologic grade 1 and grade 3 patients) and a validation set (histologic grade 2 and unknown grade patients). Gene expression values were then standardized within the interval [x_imin, x_imax]. This linear re-scaling of the variable into the interval [0,1] was performed according to equation (1) where $\widehat{x_i}$ is the i-th gene probe (feature) and ${x}_i$ is its normalized value; *x_imin* and *x_imax* are the bounds of the i-th probe for the corresponding cohort.
  1 $$ {x}_i = \frac{{\widehat{x}}_i-{{\widehat{x}}_{i\,\,\,\,\min}}}{{\widehat{x}}_{i \max }-{\widehat{x}}_{i \min }}$$
  This standardization, based only on histologic grade 1 and grade 3 status, was necessary since gene expression values in different platforms are scaled by unknown parameters and because grade compositions diverge between datasets.
2. Next, each gene probe value was *fuzzified* using the training data based on the appropriate learning process (LAMDA). The resulting fuzzy sets represent the probe’s (feature) membership to each of the two existing classes. Probe *fuzzification* was performed according to two membership functions proposed by Aguado and Aguilar in [64]. Both functions were tested in order to identify which gene subsets provided the highest discrimination power (the objective is to find the highest classification accuracy with a minimum number of probes).
3. Once all gene expression values from probes had been *fuzzified*, the fuzzy feature selection algorithm MEMBAS was performed to rank probes in descending order according to their resulting fuzzy weight (*w*_*f*_). Then, iteratively, the classification performance in terms of overall classification error, sensitivity (percentage of histologic grade 3 tumors correctly classified), and specificity (percentage of histologic grade 1 tumors correctly classified) was calculated using LAMDA classification algorithm. Hence, for each iteration, the number of probes was incremented, following the ranked order, until the whole probe set was tested. Since most cohort datasets are scarce and small (in terms of patient number), classification performance was evaluated with a leave-one-out cross-validation (LOOCV) to estimate the optimal classification parameters as proposed by Wessels *et al.* [65]. This method consists in holding out one single sample from the histologic grade 1 and grade 3 dataset of the cohort. This sample was considered as the validation data and the remaining histologic grade 1 and grade 3 samples of the cohort were used to estimate the optimal parameters of the classifier (profile of each class, exigency-*α*). The resulting model was employed to classify the test sample. This was carried out on all samples so that each of them was used once for testing. This procedure is commonly used to evaluate the generalization performance of a classifier, since it reduces bias from the training set, i.e. data for a sample (tumor) are never used to estimate the classifier parameters (class profile) for its own classification. The resulting probe set, providing the higher classification performance, characterizes the *fuzzy Molecular Grade 3-like* (*f*MG3) and *fuzzy Molecular Grade 1-like* classes.
4. Given that LAMDA classification provides a global adequacy degree (GAD) for each sample to both classes (Figure 2), a molecular grade score has been introduced in order to determine the similarity between the Gene Expression Grade and the histologic grade. This score was determined by taking the GAD of each sample (tumor) for the *Fuzzy Molecular Grade 3-like (fMG3)* class. Then, if the score was greater than or equal 0.5 the patient is most likely to have a Grade 3-like gene expression profile (*f*MG3) and if the score was less than 0.5 the patient experiences a Grade 1-like gene expression profile (*f*MG1).
5. Once the optimal probe subset had been identified for a given cohort, the histologic grade 2 and unknown grade patients were classified into either *f*MG1-like or *f*MG3-like according to their resulting score (GAD).

### NimbleGen Microarrays

Gene signatures obtained were validated on an additional cohort. In order to focus the attention to the most informative genes, custom NimbleGen Roche 4x72K gene expression microarray kit (NimbleGen Roche Diagnostics, Meylan, France) was designed by NimbleGen support, based on genes included in gene signatures provided by the proposed fuzzy logic selection strategy (2030 accession numbers). We added controls of microarray experiment and housekeeping genes for normalization data. A mean of 9 probes by sequence were designed.

### Validation cohort

In order to validate the different generated gene signatures (*f*GS), 150 frozen breast cancer tumors from the tumor bank of the Claudius Regaud Institute (ICR Toulouse, France) were selected. This cohort consisted of consecutive invasive breast carcinoma patients treated at Claudius Regaud Institute between 2009 and 2011. All patients included in this cohort signed an informed consent. Clinico-pathological characteristics of the series were similar to those observed in routine clinical practice (i.e. majority of pre-menopausal patients presenting with T1c, node negative, ER+ invasive ductal carcinoma of intermediate grade). Clinical–pathological data of our samples are available in Additional file 11: Table S8. The study was approved by the local ethic committee.

### RNA extraction and microarray experiments

Total RNA was extracted from 12 μm-thickcryostat sections of with Qiazol and RNeasy Lipid Tissue Kit (Qiagen, Courtaboeuf, France). The concentration of total RNA obtained from each tumor sample was determined using a Nanodrop® spectrophotometer (Labtech, Palaiseau, France) and the integrity of the RNA was assessed using a 2100 Bioanalyzer® (Agilent Technologies, Massy, France). The percentage of tumor cells was evaluated on frozen a section stained with hematoxylin-eosin. Double-stranded cDNA (ds-cDNA) was synthesized from 2 μg of total RNA using SuperScript One cycle kit (Invitrogen Life Technologies, Saint-Aubin, France) with random primers and Oligo(dT) primers, then cleaned with MinElute PCR Purification Kit (Qiagen, Courtaboeuf, France). ERCC RNA Spike-In Control Mix (Ambion Life technologies, Saint-Aubin, France), a set of external RNA positive controls, was added to total RNA at the beginning of the experiment to assess accuracy of measurements of gene expression. One μg ds-cDNA was Cy3 labeled using One-Color DNA labeling kit (NimbleGen Roche Diagnostics, Meylan, France). Hybridization on our Roche NimbleGen 4x72K custom microarray, washing slides and scan were performed according to the manufacturer’s protocol (NimbleGen Roche Diagnostics, Meylan, France). DEVA software v1.2 (NimbleGen Roche Diagnostics, Meylan, France) was used to extract the raw intensity values. Spike in control analyses, normalization and statistical analyses of the data were performed with Bioconductor packages (http://www.bioconductor.org/) and R software (version 2.14.1). All data were Robust Multichip Average (RMA) background corrected, log2-transformed, summarized by a robust mean for each probe. Then, housekeeping normalization was done on the experimental data. This method involved subtracting the average from a subset of 20 selected reference genes. As numerous studies reported that commonly used reference genes are not constantly expressed under different experimental conditions, we selected the 20 most suitable reference genes among 62 candidate genes previously reported as ‘housekeeping’ genes, based on their expression stability and correlation. Correlation coefficient between fluorescence and quantity of controls were comprised from 0.96 to 1, providing high confidence in the experimental data. The microarray data have been deposited in the Gene Expression Omnibus (GEO) public database. The GSE53958 study can be found at: http://www.ncbi.nlm.nih.gov/geo/query/acc.cgi?acc=GSE53958.

### Survival analysis

Disease-free survival was defined as the time interval from surgery until any type of recurrence (local, regional, or distant) or last date of follow-up [54]. Survival rates were estimated by the Kaplan-meier methods and comparison between groups were performed using Logrank test. Using cox proportional hazard modeling, hazard ratios were estimated with their corresponding 95% confidence interval.

All P-values reported were two-sided. For all statistical tests, differences were considered significant at the 5% level. Statistical analyses were performed using R software.

## Additional files

Additional file 1: Table S1. Overlapped genes between GSs. Additional file 2: Table S2. Validation tests; Agreement in classification between molecular and histologic grades in validation cohorts. Additional file 3: Figure S1. Relapse free survival analysis of patients with histologic grade 1 (−−---) and 3 (......) tumors classified in fMG1-like (green) and fMG3-like (red) by fuzzy Gene Signatures (fGS) in NKI2, KJX64/KJ125, Transbig, Stockholm cohorts. Hazard ratios with 95% confidence intervals (CI) and log-rank test (p value) were calculated to evaluate significance (fMG1-like vs. fMG3-like and HG1 vs. HG3). (A) fGS n° A. (B) fGS n° B. (C) fGS n° C. (D) fGS n° D. Additional file 4: Table S3. Survival analysis of grade 2 tumors separated in grade 1-like and grade 3-like according to their molecular grade score. Additional file 5: Table S4. Univariate and multivariate analysis of breast cancer prognostic factors for the *f*GS B (n = 118). Additional file 6: Table S5. Agreement in classification between molecular and histologic grades in C. Regaud cohort. Additional file 7: Table S6. Selected NimbleGen’ probes for the four gene signatures. Additional file 8: Figure S2. Venn diagram showing the overlap between the four new gene signatures fGS A, fGS B, fGS C and fGS D obtained when we applied the fuzzy logic selection on breast cancer microarray databases. Additional file 9: Table S7. Microarray datasets used for gene signature generation: distribution of patients according to clinical variables. Additional file 10:
**Including mathematical detail of both feature selection (MEMBAS) and classification (LAMDA) algorithms.**
Additional file 11: Table S8. Patients’ characteristics from Claudius Regaud Institute.
