# Supplementary material for: Fuzzy logic selection as a new reliable tool to identify molecular grade signatures in breast cancer – the INNODIAG study
Source: BMC Med Genomics. 2015 Feb 7;8:3. doi: 10.1186/s12920-015-0077-1 (PMC4342216; doi:10.1186/s12920-015-0077-1)
Supplement: Additional file 1: Table S1. — Overlapped genes between GSs. [file 12920_2015_77_MOESM1_ESM.pdf]

	Number of genes in GS	Overlapped genes between GSs			
		*f*GS A [16]	*f*GS B [14]	*f*GS C [13]	*f*GS D [6]
Fuzzy Gene signature (*f*GS) A [16]	65	65	8	21	5
Fuzzy Gene signature (*f*GS) B [14]	37	8	37	27	8
Fuzzy Gene signature (*f*GS) C [13]	65	21	27	65	15
Fuzzy Gene signature (*f*GS) D [6]	16	5	8	15	16
21-gene Recurrence Score (Paik *et al.*) [18]	21	3	4	5	2
PAM genetic grade signature (Ivshina *et al.*) [13]	18	3	5	14	5
Genomic Grade Index (Sotiriou et al.) [14]	111	27	33	50	15
76-gene signature (Wang *et al.*) [17]	76	1	1	1	0
70-gene signature (Van't Veer *et al.*) [15]	68	6	3	5	2
Molecular grade index and 2-gene HOXB13;IL17BR (Ma *et al.*) [19]	7	3	3	4	2
