# Supplementary material for: Fuzzy logic selection as a new reliable tool to identify molecular grade signatures in breast cancer – the INNODIAG study
Source: BMC Med Genomics. 2015 Feb 7;8:3. doi: 10.1186/s12920-015-0077-1 (PMC4342216; doi:10.1186/s12920-015-0077-1)
Supplement: Additional file 2: Table S2. — Validation tests; Agreement in classification between molecular and histologic grades in validation cohorts. [file 12920_2015_77_MOESM2_ESM.pdf]

Dataset	Molecular grade	Histologic grade											
		fGS B				*f* GS C				*f* GS D			
		HG1		HG3		HG1		HG3		HG1		HG3	
		n	%	n	%	n	%	n	%	n	%	n	%
KJX64/KJ125 (n=126)	*f* MG1									**57**	**(85%)**	8	(14%)
GSE2990	*f* MG3									10	(15%)	**51**	**(86%)**
Uppsala (n=123)	*f* MG1									**64**	**(94%)**	5	(9%)
GSE4922	*f* MG3									4	(6%)	**50**	**(91%)**
Transbig (n=113)	*f* MG1	**26**	**(87%)**	15	(18%)								
GSE7390	*f* MG3	4	(13%)	**68**	**(82%)**								
Stockholm (n=89)	*f* MG1	**24**	**(86%)**	8	(13%)	**24**	**(86%)**	9	(15%)	**23**	**(82%)**	7	(11%)
GSE1456	*f* MG3	4	(14%)	**53**	**(87%)**	4	(14%)	**52**	**(85%)**	5	(18%)	**54**	**(89%)**
