# Supplementary figures and images for: Fuzzy logic selection as a new reliable tool to identify molecular grade signatures in breast cancer – the INNODIAG study
Source: BMC Med Genomics. 2015 Feb 7;8:3. doi: 10.1186/s12920-015-0077-1 (PMC4342216; doi:10.1186/s12920-015-0077-1)

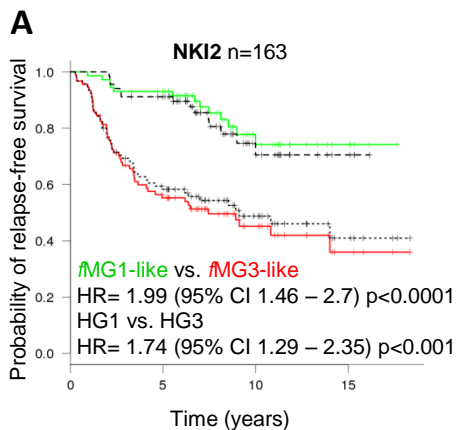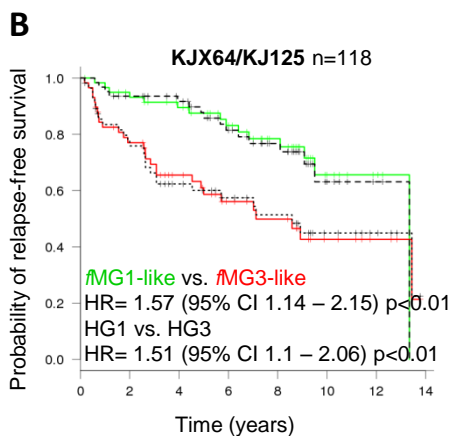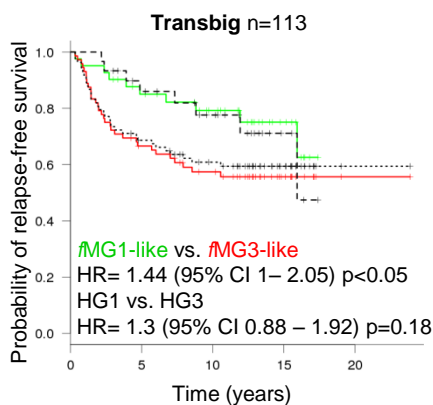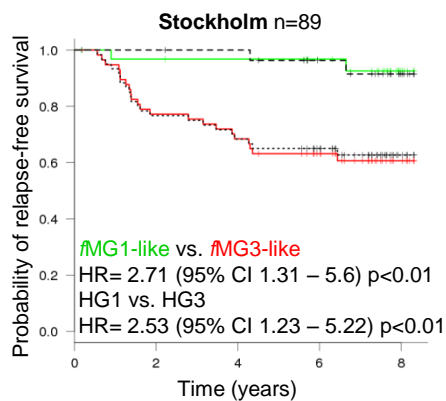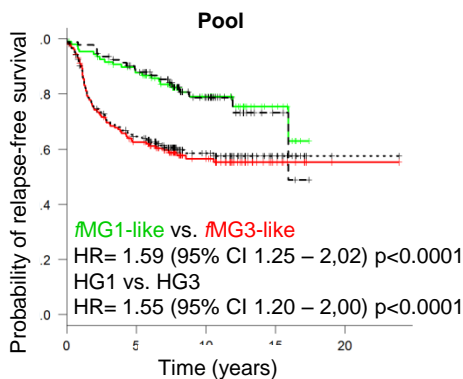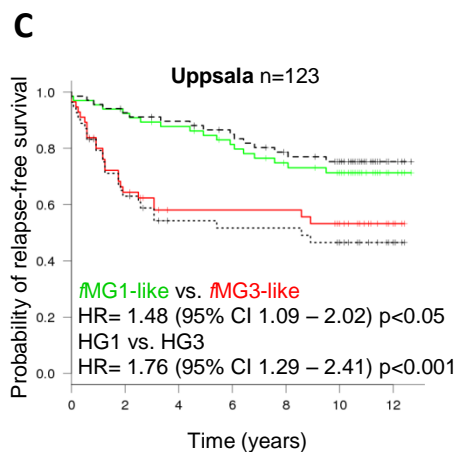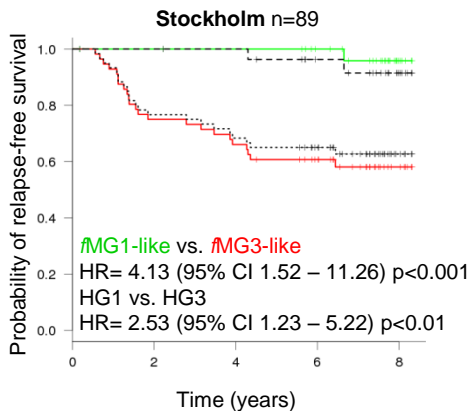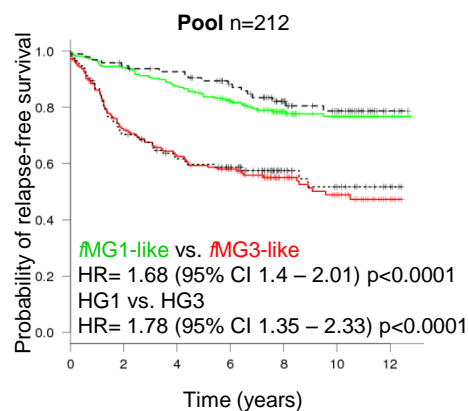

**D**

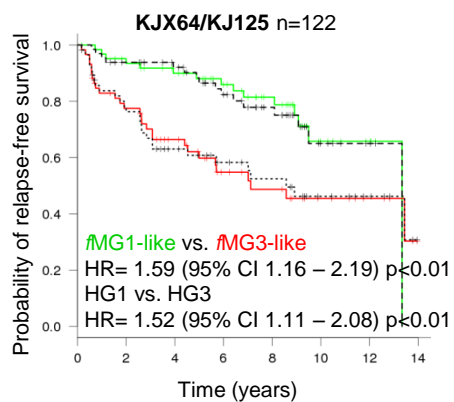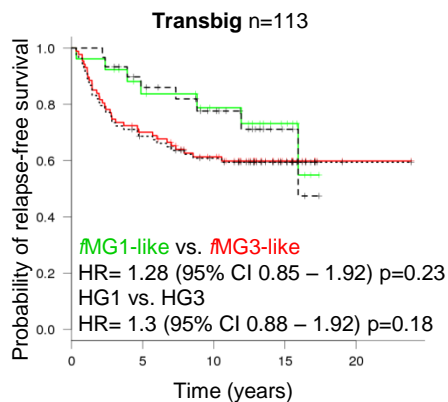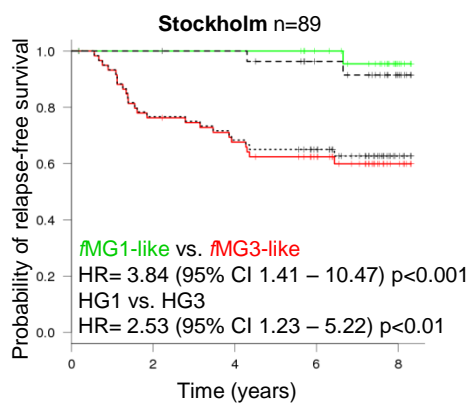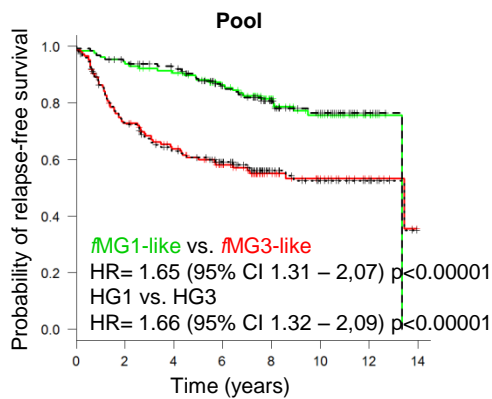

Supplement: Additional file 3: Figure S1. — Relapse free survival analysis of patients with histologic grade 1 (−−---) and 3 (......) tumors classified in fMG1-like (green) and fMG3-like (red) by fuzzy Gene Signatures (fGS) in NKI2, KJX64/KJ125, Transbig, Stockholm cohorts. Hazard ratios with 95% confidence intervals (CI) and log-rank test (p value) were calculated to evaluate significance (fMG1-like vs. fMG3-like and HG1 vs. HG3). (A) fGS n° A. (B) fGS n° B. (C) fGS n° C. (D) fGS n° D. [file 12920_2015_77_MOESM3_ESM.pdf]

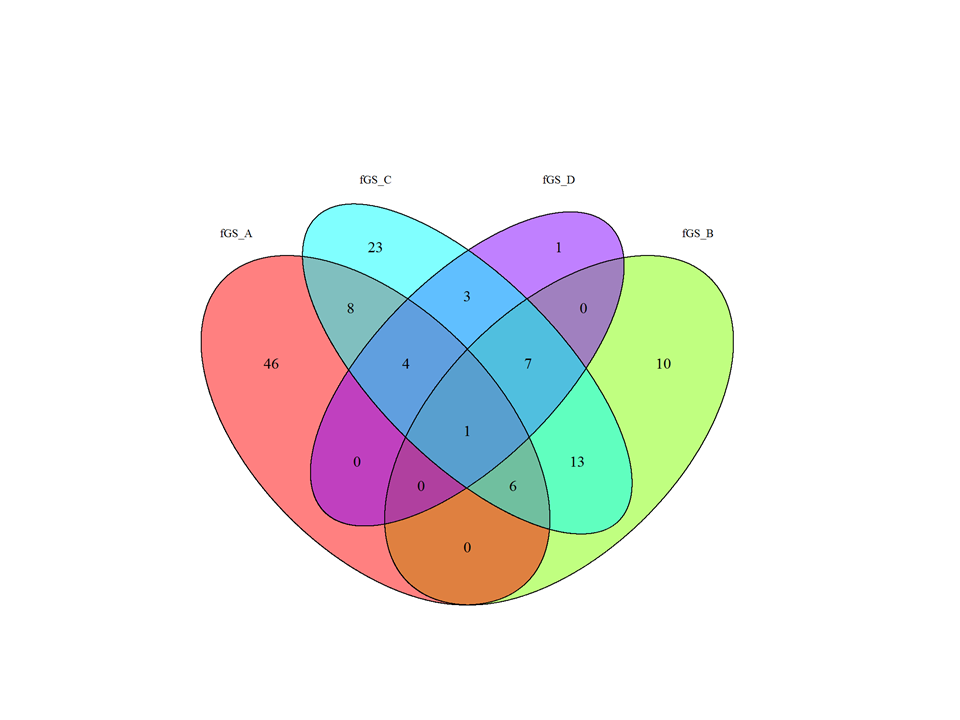

Supplement: Additional file 8: Figure S2. — Venn diagram showing the overlap between the four new gene signatures fGS A, fGS B, fGS C and fGS D obtained when we applied the fuzzy logic selection on breast cancer microarray databases. [file 12920_2015_77_MOESM8_ESM.tiff]
