# Supplementary material for: Fuzzy logic selection as a new reliable tool to identify molecular grade signatures in breast cancer – the INNODIAG study
Source: BMC Med Genomics. 2015 Feb 7;8:3. doi: 10.1186/s12920-015-0077-1 (PMC4342216; doi:10.1186/s12920-015-0077-1)
Supplement: Additional file 4: Table S3. — Survival analysis of grade 2 tumors separated in grade 1-like and grade 3-like according to their molecular grade score. [file 12920_2015_77_MOESM4_ESM.pdf]

***fuzzy* Gene signature A**			
NKI2	93	1.406 (1.004 - 1.969)	p<0.043
***fuzzy* Gene signature B**			
Pool	309	1.76 (1.46 - 2.11)	p<0.0001
KJ125	63	1.72 (1.137 - 2.602)	p<0.0073
Uppsala	126	1.553 (1.166 - 2.068)	p<0.002
Transbig	83	1.984 (1.466 - 2.685)	$p<1.94x10^{-06}$
Stockholm	58	1.809 (1.046 - 3.128)	p<0.0249
***fuzzy* Gene signature C**			
Pool	184	1.687 (1.311 - 2.171)	$p<2.1x10^{-05}$
Uppsala	126	1.571 (1.18 - 2.092)	p<0.00139
Stockholm	58	2.128 (1.231 - 3.677)	p<0.004
***fuzzy* Gene signature D**			
Pool	309	1.53 (1.27 - 1.83)	p<0.0001
KJ125	63	1.581 (1.052 - 2.376)	p<0.0229
Uppsala	126	1.431 (1.071 - 1.911)	p<0.0135
Transbig	83	1.497 (1.085 - 2.064)	p<0.0117
Stockholm	58	1.587 (0.888 - 2.837)	p=0.107
