# Supplementary material for: Fuzzy logic selection as a new reliable tool to identify molecular grade signatures in breast cancer – the INNODIAG study
Source: BMC Med Genomics. 2015 Feb 7;8:3. doi: 10.1186/s12920-015-0077-1 (PMC4342216; doi:10.1186/s12920-015-0077-1)
Supplement: Additional file 5: Table S4. — Univariate and multivariate analysis of breast cancer prognostic factors for the fGS B (n = 118). [file 12920_2015_77_MOESM5_ESM.pdf]

	Univariate analysis		Multivariable analysis	
Comparison	HR (95% CI) †	P‡	HR (95% CI) †	P‡
**Age**				
=< 50 vs > 50	0.89 (0.69 to 1.16)	p=0.3996	0.86 (0.65 to 1.14)	p=0.283865
**Histological Grade**				
G2 + G3 vs G1	1.93 (1.37 to 2.73)	p=8.702e-05	1.31 (0.89 to 1.93)	p=0.374097
G3 vs G1 + G2	1.45 (1.11 to 1.90)		0.65 (0.46 to 0.92)	
**Estrogen receptor status**				
positive vs negative	0.75 (0.56 to 1.01)	p=0.05596	1.12 (0.80 to 1.57)	p=0.498064
**Lymphome node status**				
positive vs negative	1.54 (1.12 to 2.12)	p=0.007772	1.39 (0.99 to 1.96)	p=0.05691
**Tumor size**				
>2 cm vs =< 2 cm	1.92 (1.48 to 2.49)	p=1.074e-06	1.64 (1.24 to 2.17)	p=0.000479
**fGS B**				
G1 vs G3	1.53 (1.35 to 1.74)	p=1.309e-10	1.51 (1.21 to 1.88)	p=0.000320

† HR = hazard ratio for recurrence; CI = confidence interval.
‡ *P. Value* based on Cox regression.
