# Supplementary material for: Fuzzy logic selection as a new reliable tool to identify molecular grade signatures in breast cancer – the INNODIAG study
Source: BMC Med Genomics. 2015 Feb 7;8:3. doi: 10.1186/s12920-015-0077-1 (PMC4342216; doi:10.1186/s12920-015-0077-1)
Supplement: Additional file 6: Table S5. — Agreement in classification between molecular and histologic grades in C. Regaud cohort. [file 12920_2015_77_MOESM6_ESM.pdf]

fuzzy Gene signature	Molecular grade	Histologic grade					
		G1		G3		G2	
		n	%	n	%	n	%
f GS A	G1	**14**	**(78%)**	5	(7%)	33	(57%)
	G3	4	(22%)	**69**	**(93%)**	25	(43%)
f GS B	G1	**17**	**(94%)**	3	(4%)	37	(64%)
	G3	1	(6%)	**71**	**(96%)**	21	(36%)
f GS C	G1	**14**	**(78%)**	3	(4%)	23	(40%)
	G3	4	(22%)	**71**	**(96%)**	35	(60%)
f GS D	G1	**17**	**(94%)**	4	(5%)	31	(53%)
	G3	1	(6%)	**70**	**(95%)**	27	(47%)
