# Supplementary material for: Fuzzy logic selection as a new reliable tool to identify molecular grade signatures in breast cancer – the INNODIAG study
Source: BMC Med Genomics. 2015 Feb 7;8:3. doi: 10.1186/s12920-015-0077-1 (PMC4342216; doi:10.1186/s12920-015-0077-1)
Supplement: Additional file 7: Table S6. — Selected NimbleGen’ probes for the four gene signatures. [file 12920_2015_77_MOESM7_ESM.pdf]

PROBE_ID	Seq_ID	Gene Symbol	NB GS overlapped	ƒGS A	ƒGS B	ƒGS C	ƒGS D
NM_001042426P00257	NM_001042426	CENPA	4	1	1	1	1
NM_001168P00369	NM_001168	BIRC5	3	1	1	1	0
NM_004336P01393	NM_004336	BUB1	3	1	0	1	1
NM_031966P01100	NM_031966	CCNB1	3	1	1	1	0
NM_004701P00690	NM_004701	CCNB2	3	1	0	1	1
NM_005733P00981	NM_005733	KIF20A	3	1	1	1	0
NM_002266P00573	NM_002266	KPNA2	3	1	1	1	0
NM_015341P00263	NM_015341	NCAPH	3	1	0	1	1
NM_001126103P01120	NM_001126103	RACGAP1	3	1	1	1	0
NM_001034P00871	NM_001034	RRM2	3	1	0	1	1
NM_181800P00113	NM_181800	UBE2C	3	1	1	1	0
NM_003600P00703	NM_003600	AURKA	3	0	1	1	1
NM_018101P00402	NM_018101	CDCA8	3	0	1	1	1
NR_038336P00896	NR_038336	DDX39A	3	0	1	1	1
NM_202002P00073	NM_202002	FOXM1	3	0	1	1	1
NM_006845P00538	NM_006845	KIF2C	3	0	1	1	1
NM_014791P00543	NM_014791	MELK	3	0	1	1	1
NM_001145966P00680	NM_001145966	MKI67	3	0	1	1	1
NM_031299P00260	NM_031299	CDCA3	2	1	0	1	0
NM_012291P05499	NM_012291	ESPL1	2	1	0	1	0
NM_018410P00571	NM_018410	HJURP	2	1	0	1	0
NM_014875P01721	NM_014875	KIF14	2	1	0	1	0
NM_002263P00879	NM_002263	KIFC1	2	1	0	1	0
NM_199414P01168	NM_199414	PRC1	2	1	0	1	0
NM_006461P03417	NM_006461	SPAG5	2	1	0	1	0
NM_014501P00036	NM_014501	UBE2S	2	1	0	1	0
NM_018136P01738	NM_018136	ASPM	2	0	1	1	0
NM_001211P01494	NM_001211	BUB1B	2	0	1	1	0
NM_033379P00154	NM_033379	CDK1	2	0	1	1	0
NM_001130851P00059	NM_001130851	CDKN3	2	0	1	1	0
NM_014750P00453	NM_014750	DLGAP5	2	0	1	1	0
NM_004523P00083	NM_004523	KIF11	2	0	1	1	0
NM_012310P00482	NM_012310	KIF4A	2	0	1	1	0
NM_002358P00387	NM_002358	MAD2L1	2	0	1	1	0
NM_004219P00350	NM_004219	PTTG1	2	0	1	1	0
NM_003486P01279	NM_003486	SLC7A5	2	0	1	1	0
NM_003714P00728	NM_003714	STC2	2	0	1	1	0
NM_012112P00190	NM_012112	TPX2	2	0	1	1	0
NM_004237P00195	NM_004237	TRIP13	2	0	1	1	0
NM_001255P01049	NM_001255	CDC20	2	0	0	1	1
NM_001127182P01178	NM_001127182	CEP55	2	0	0	1	1
NM_018518P01731	NM_018518	MCM10	2	0	0	1	1

PROBE_ID	Seq_ID	Gene Symbol	NB GS overlapped	ƒGS A	ƒGS B	ƒGS C	ƒGS D
NM_001135704P00755	NM_001135704	ACBD4	1	1	0	0	0
NM_018209P00463	NM_018209	ARFGAP1	1	1	0	0	0
NM_004217P00574	NM_004217	AURKB	1	1	0	0	0
NM_000060P01573	NM_000060	BTD	1	1	0	0	0
NM_152259P03283	NM_152259	C15orf42	1	1	0	0	0
NM_001789P01139	NM_001789	CDC25A	1	1	0	0	0
NM_003504P00461	NM_003504	CDC45	1	1	0	0	0
NM_016343P04954	NM_016343	CENPF	1	1	0	0	0
NR_023313P00440	NR_023313	CIRBP	1	1	0	0	0
NM_053277P01727	NM_053277	CLIC6	1	1	0	0	0
NM_021110P01436	NM_021110	COL14A1	1	1	0	0	0
NM_001565P00039	NM_001565	CXCL10	1	1	0	0	0
NM_016448P02128	NM_016448	DTL	1	1	0	0	0
NM_005225P00707	NM_005225	E2F1	1	1	0	0	0
NM_004091P00765	NM_004091	E2F2	1	1	0	0	0
NM_001402P00630	NM_001402	EEF1A1	1	1	0	0	0
NM_033255P00372	NM_033255	EPSTI1	1	1	0	0	0
NM_003686P01331	NM_003686	EXO1	1	1	0	0	0
NM_002001P00689	NM_002001	FCER1A	1	1	0	0	0
NM_002053P01089	NM_002053	GBP1	1	1	0	0	0
NM_005342P00165	NM_005342	HMGB3	1	1	0	0	0
NM_001002032P00278	NM_001002032	HN1	1	1	0	0	0
NM_031266P00144	NM_031266	HNRNPAB	1	1	0	0	0
AI765936P00045	AI765936	HS cDNA	1	1	0	0	0
NM_005101P00177	NM_005101	ISG15	1	1	0	0	0
NM_015254P04419	NM_015254	KIF13B	1	1	0	0	0
NM_006983P01020	NM_006983	MMP23B	1	1	0	0	0
NM_001144925P00001	NM_001144925	MX1	1	1	0	0	0
NM_001243144P00551	NM_001243144	NUSAP1	1	1	0	0	0
NM_002535P00730	NM_002535	OAS2	1	1	0	0	0
NR_037620P00843	NR_037620	ORC6	1	1	0	0	0
NM_152341P00001	NM_152341	PAQR4	1	1	0	0	0
NM_138325P01605	NM_138325	PCSK6	1	1	0	0	0
NM_001100877P00640	NM_001100877	PHYHD1	1	1	0	0	0
NM_001168357P00849	NM_001168357	PLA2G7	1	1	0	0	0
NM_006607P00350	NM_006607	PTTG2	1	1	0	0	0
NR_002734P00350	NR_002734	PTTG3P	1	1	0	0	0
NM_020954P00920	NM_020954	RNF213	1	1	0	0	0
NM_020974P00110	NM_020974	SCUBE2	1	1	0	0	0
NM_001045P01394	NM_001045	SLC6A4	1	1	0	0	0
NM_004787P00008	NM_004787	SLIT2	1	1	0	0	0
NM_001145673P00001	NM_001145673	SORBS2	1	1	0	0	0
NM_013443P00883	NM_013443	ST6GALNAC6	1	1	0	0	0
NM_003258P00230	NM_003258	TK1	1	1	0	0	0
NM_003295P00170	NM_003295	TPT1	1	1	0	0	0
NM_017414P00616	NM_017414	USP18	1	1	0	0	0
NM_175709P00664	NM_175709	CBX7	1	0	1	0	0
NM_001237P00750	NM_001237	CCNA2	1	0	1	0	0
NM_006733P00910	NM_006733	CENPI	1	0	1	0	0
NM_024053P00218	NM_024053	CENPM	1	0	1	0	0
NM_001171171P00454	NM_001171171	CX3CR1	1	0	1	0	0
NM_001198961P00322	NM_001198961	ECHDC2	1	0	1	0	0
NM_004111P00542	NM_004111	FEN1	1	0	1	0	0
NM_001142557P00877	NM_001142557	HMMR	1	0	1	0	0
NM_001198557P00286	NM_001198557	LMNB1	1	0	1	0	0
NM_006101P00257	NM_006101	NDC80	1	0	1	0	0
NM_018685P02179	NM_018685	ANLN	1	0	0	1	0
NM_000057P03458	NM_000057	BLM	1	0	0	1	0
NM_001160138P01055	NM_001160138	C7orf63	1	0	0	1	0
NM_057749P00356	NM_057749	CCNE2	1	0	0	1	0
NM_001813P03818	NM_001813	CENPE	1	0	0	1	0
NM_001100625P00197	NM_001100625	CENPN	1	0	0	1	0
NM_001012507P00112	NM_001012507	CENPW	1	0	0	1	0
NM_004456P01117	NM_004456	EZH2	1	0	0	1	0
NM_005252P00001	NM_005252	FOS	1	0	0	1	0
NM_021067P00195	NM_021067	GINS1	1	0	0	1	0
NM_031217P00745	NM_031217	KIF18A	1	0	0	1	0
NM_018407P00687	NM_018407	LAPTM4B	1	0	0	1	0
NM_005910P00873	NM_005910	MAPT	1	0	0	1	0
NM_005915P01449	NM_005915	MCM6	1	0	0	1	0
NM_020409P00138	NM_020409	MRPL47	1	0	0	1	0
NM_015969P00205	NM_015969	MRPS17	1	0	0	1	0
NM_002466P00463	NM_002466	MYBL2	1	0	0	1	0
NM_031423P01107	NM_031423	NUF2	1	0	0	1	0
NM_004203P00851	NM_004203	PKMYT1	1	0	0	1	0
NM_018304P00787	NM_018304	PRR11	1	0	0	1	0
NM_001126044P00578	NM_001126044	PTGER3	1	0	0	1	0
NM_001032283P00642	NM_001032283	TMPO	1	0	0	1	0
NM_003318P01204	NM_003318	TTK	1	0	0	1	0
NM_001166061P00853	NM_001166061	GLRB	1	0	0	0	1
