# Supplementary material for: Fuzzy logic selection as a new reliable tool to identify molecular grade signatures in breast cancer – the INNODIAG study
Source: BMC Med Genomics. 2015 Feb 7;8:3. doi: 10.1186/s12920-015-0077-1 (PMC4342216; doi:10.1186/s12920-015-0077-1)
Supplement: Additional file 9: Table S7. — Microarray datasets used for gene signature generation: distribution of patients according to clinical variables. [file 12920_2015_77_MOESM9_ESM.pdf]

**Dataset**	**NKI2**	**KJX64**	**KJ125**	**Uppsala**	**Transbig**
Microarray platform	Agilent	Affymetrix U133A		Affymetrix U133 A & B	Affymetrix U133A
Reference	Van de Vijver *et al.* [16]	Sotiriou *et al.* [14]		Ivshina *et al.* [13]	Desmedt *et al.* [6]
Source	bioinformatics.nki.nl/data.php	GSE2990		GSE4922	GSE7390
No. of samples	256	64	125	249	198
ER status, No.					
Negative	60	0	34	34	64
Positive	196	64	85	211	134
N/A	0	0	6	4	0
Histological grade, No.					
1	68	33	34	68	30
2	93	0	46	126	83
3	95	31	28	55	83
N/A	0	0	17	0	2
Lymph node status, No.					
Negative	116	28	125	159	198
Positive	140	30	0	81	0
N/A	0	6	0	9	0
Systemic treatment	yes and no	yes	no	yes and no	no
