# Supplementary material for: Fuzzy logic selection as a new reliable tool to identify molecular grade signatures in breast cancer – the INNODIAG study
Source: BMC Med Genomics. 2015 Feb 7;8:3. doi: 10.1186/s12920-015-0077-1 (PMC4342216; doi:10.1186/s12920-015-0077-1)
Supplement: Additional file 10: — Including mathematical detail of both feature selection (MEMBAS) and classification (LAMDA) algorithms. [file 12920_2015_77_MOESM10_ESM.pdf]

Supplementary Information File

Fuzzy Molecular Grade Signatures

Memebership functions

To transform quantitative data (features) values into the unified membership space several membership functions denoted as μ_k^i can be used [62].

- "Fuzzy" extension of the binomial function:

$$\mu_k^i\left[x_i \middle| \varphi_k^i\right] = {\varphi_k^i}^{x_i}\left(1-\varphi_k^i\right)^{1-x_i} \tag{1}$$

where φ_k^i corresponds to the mean value for the ith feature characterizing class k. This membership function works extremely well when the observations are grouped, after standardization, around 0 or 1, but may present instability or definition problems when data are concentrated around 0.5. Therefore, a membership function which includes the proximity to an estimated center should also be considered. Moreover, when the volume of the observed data is important, it is very likely to follow a Gaussian or semi-Gaussian distribution.

- Gaussian function:

$$\mu_k^i\left[x_i \middle| \varphi_k^i, \sigma_k^i\right] = e^{-\frac{1}{2}\frac{\left(x_i-\varphi_k^i\right)^2}{{\sigma_k^i}^2}} \tag{2}$$

where, φ_k^i is the mean value, and parameter σ_k^i measures the proximity (variance) of the ith feature values based on the samples belonging to class C_k.

Fuzzy feature selection algorithm - MEMBAS (binary class problems)

Assuming that the nth data sample $x_n = [x_n^1,\ x_n^2,\ \cdots, x_n^m]$ is labeled by class *c*, let $\tilde{c}$ be the alternative class, the membership margin for sample x_n is defined by:

$$\beta_n = \psi(U_{nc}) - \psi(U_{n\tilde{c}}) \quad (3)$$

Where U_{nc} and $U_{n\tilde{c}}$ are respectively the membership degree vectors of sample x_n to classes c and $\tilde{c}$, $\psi(Y) = \sum_i Y_i$ computes the global contribution of a subset of features to each class. Note that sample x_n is correctly classified if $\beta_n > 0$.

The basic idea is to determine the fuzzy feature weights (w_f) which minimize the leave-one-out error:

$$\underset{w_f}{\text{Max}} \sum_{n=1}^{N} \beta_n(w_f) = \sum_{n=1}^{N} \{\sum_{i=1}^{m} w_{fi}\mu_c^i(x_{ni}) - \sum_{i=1}^{m} w_{fi}\mu_{\tilde{c}}^i(x_{ni})\} \ \text{S.t}\ \|w_f\|_2^2 = 1,\ w_f \geq 0 \quad (4)$$

where $\beta_n(w_f)$ is the margin of x_n computed with respect to w_f. The first constraint is the standardized bound for the modulus of w_f so that the maximization ends up with non-infinite values, whereas the second guarantees the non-negative weight property.

By using the classical Lagrangian optimization approach an analytical solution can be derived and the solution can be written in a closed-form as:

$$w_f^* = \frac{s^+}{\|s^+\|} \ \text{where}\ \ s = \sum_{n=1}^{N} \{U_{nc} - U_{n\tilde{c}}\} \ \text{with}\ \ s^+ = [\max(s_1,0),\ \ldots,\ \max(s_m,0)]^T \quad (5)$$

In this way features are rank-ordered according to their discriminant power (w_f). Therefore, MEMBAS chooses only the features if they contribute to the overall performance. Hence, it addresses the issues of features correlation and redundancy. In Hedjazi [REF These de Lyamine] an extensive experimental study, including a comparison with known feature selection methods has been performed on several datasets presenting mixed-type and high-dimensional data.

Fuzzy classification algorithm – LAMDA

LAMDA (Learning Algorithm for Multivariable Data Analysis) [56], is a fuzzy methodology of conceptual clustering and classification. It is based on finding the global membership degree of a sample to an existing class, considering all the contributions of each feature. This contribution is called the marginal adequacy degree (MAD). To calculate the marginal contributions the same membership functions μ_k^i proposed by (Aguado and Aguilar-Martin, 1999) can be used. In this work the "fuzzy" extension of the binomial function(1) and the Gaussian function (2) were used.

The MADs are combined using "fuzzy mixed connectives" as aggregation operators in order to obtain the global adequacy degree (GAD) of an element to a class [57].

Fuzzy logic connectives are fuzzy versions of the binary logic operators, particularly, intersection (*t-norm*) and union (*t-conorm*). The aggregation function is a linear interpolation

between *t-norm* (γ) and *t-conorm* (ω) as shown in Eq.6) where the parameter $\alpha, 0 \leq \alpha \leq 1$, is called exigency.

$$GAD(x_n|C) = \alpha \cdot \gamma\left(MAD(x_n^1|C), \dots, MAD(x_n^m|C)\right) + (1-\ \alpha) \cdot \omega\left(MAD(x_n^1|C), \dots, MAD(x_n^m|C)\right) \quad (6)$$
