# Supplementary material for: Fuzzy logic selection as a new reliable tool to identify molecular grade signatures in breast cancer – the INNODIAG study
Source: BMC Med Genomics. 2015 Feb 7;8:3. doi: 10.1186/s12920-015-0077-1 (PMC4342216; doi:10.1186/s12920-015-0077-1)
Supplement: Additional file 11: Table S8. — Patients’ characteristics from Claudius Regaud Institute. [file 12920_2015_77_MOESM11_ESM.pdf]

Variable		C. Regaud Cohort
Age years, median (range)		59 (29-86)
Tumor size mm, median (range)		20 (7-65)
N+, No. (%)		77 (52.7%)
LVI+, No. (%)		62 (43.1%)
Histological type, No. (%)		
	IDC - NST	112 (74.7%)
	ILC	20 (13.3%)
	other	18 (12%)
Histological grade, No. (%)		
	1	18 (11.9%)
	2	58 (38.7%)
	3	74 (49.3%)
Mitotic score, No. (%)		
	1	46 (30.7%)
	2	31 (20.7%)
	3	73 (48.6%)
ER+, No. (%)		126 (84%)
PR+, No. (%)		107 (71.3%)
HER2+, No. (%)		15 (10%)
Triple-negative, No. (%)		18 (12%)
